# Optimizing energy for a ‘green’ vaccine supply chain

**DOI:** 10.1016/j.vaccine.2014.10.053

**Published:** 2015-02-11

**Authors:** John Lloyd, Steve McCarney, Ramzi Ouhichi, Patrick Lydon, Michel Zaffran

**Affiliations:** a France; b USA; c Tunisia; d WHO, Geneva

**Keywords:** Vaccines, Supply, Coldchain, Energy, Solar, Immunization

## Abstract

This paper describes an approach piloted in the Kasserine region of Tunisia to increase the energy efficiency of the distribution of vaccines and temperature sensitive drugs. The objectives of an approach, known as the ‘net zero energy’ (NZE) supply chain were demonstrated within the first year of operation. The existing distribution system was modified to store vaccines and medicines in the same buildings and to transport them according to pre-scheduled and optimized delivery circuits. Electric utility vehicles, dedicated to the integrated delivery of vaccines and medicines, improved the regularity and reliability of the supply chains. Solar energy, linked to the electricity grid at regional and district stores, supplied over 100% of consumption meeting all energy needs for storage, cooling and transportation. Significant benefits to the quality and costs of distribution were demonstrated. Supply trips were scheduled, integrated and reliable, energy consumption was reduced, the recurrent cost of electricity was eliminated and the release of carbon to the atmosphere was reduced. Although the initial capital cost of scaling up implementation of NZE remain high today, commercial forecasts predict cost reduction for solar energy and electric vehicles that may permit a step-wise implementation over the next 7–10 years.

Efficiency in the use of energy and in the deployment of transport is already a critical component of distribution logistics in both private and public sectors of industrialized countries. The NZE approach has an intensified rationale in countries where energy costs threaten the maintenance of public health services in areas of low population density. In these countries where the mobility of health personnel and timely arrival of supplies is at risk, NZE has the potential to reduce energy costs and release recurrent budget to other needs of service delivery while also improving the supply chain.

## Highlights

• High energy efficiency in storing and delivering vaccines reduced the recurrent costs of distribution.
• Adopting a system of planned vaccine deliveries by dedicated electric vehicle was more reliable and timely.
• Solar modules on the roofs of stores linked to the electrical grid, generate enough ‘green’ energy for storage and transport.
• ‘Green’ distribution systems for vaccines and medicines meet an increasing need for cooling in public health programmes.

## 1 Introduction

Maintaining the vaccine supply chain from manufacturer to population in need is vital for the success of immunization programmes and requires highly efficient systems and technologies to be sustained, especially in resource-challenged areas. The process requires that vaccines are kept cool in a cold chain at all times during storage and transport from the national warehouse up to remote health centres. High performing and efficient distribution, combined with rigorous vaccine handling are already critical success factors in the best existing systems of global distribution.

However, many countries still suffer from chronic vaccine distribution failures due to irregular and unreliable shipments. In these countries access to transport is uncertain and poorly planned and vaccine storage conditions are not continuously monitored [1]. As the number, volume and value of vaccines and temperature-sensitive medicines steadily increases globally, the need to address supply chain challenges in these countries becomes increasingly urgent.

‘Net zero energy’ (NZE) is a concept to raise energy efficiency of buildings and processes by reducing energy consumption and waste to the minimum and by generating sufficient renewable energy on-site to offset the consumption of grid electricity (grid-linked) [2]. In this case NZE was applied as the lead concept in a selection of managerial interventions to streamline the storage and transport of vaccines and medicines of ‘net zero energy’ (NZE).

In Tunisia, a country where sun is in abundance and energy costs are escalating, where switching to renewable energy is a national priority and where the solar industry is growing, the Ministry of Health readily engaged in project Optimize [3] proposition to pilot the benefits of applying the NZE approach to the Tunisian vaccine supply chain.

The demonstration in Tunisia installed enough roof-mounted solar modules to offset the consumption of electricity (light, heat, space cooling, computing and refrigerated storage) from the grid at regional and district stores including the charging of all-electric utility vehicles overnight. The NZE approach optimized efficiency of handling supplies by physically integrating storage and transport for vaccines and medicines and relocating the stores under one roof. Dedicated use of the electric vehicle for pre-planned supply trips assured regularity and reliability while also reducing recurrent energy cost.

The aim of this paper is to present evidence from a demonstration in the south-western part of Tunisia (2012) on the value proposition of a ‘green’ vaccine supply chain for the future using the ‘NZE’ approach.

## 2 Method

In 2012 and under the auspices of project Optimize [4], an innovative demonstration was conducted in the region of Kasserine, Tunisia, in the municipality and in three rural districts within the region.^1^ The project aimed to demonstrate a highly energy efficient storage and delivery system for vaccines and medicines based on the NZE concept of ‘net zero energy’ (NZE). The concept minimizes harmful effects on the environment by cutting carbon emissions and it optimizes the production and consumption of energy by the use of renewable energy in tandem with the electricity grid. This application of the NZE concept is set in the context of a selection of interventions that together streamline the distribution system.

The methodology of the demonstration proceeded in four stages.

### 2.1 Selection of demonstration areas

Out of the 24 regions of Tunisia, the Ministry of Health selected Kasserine to conduct an NZE demonstration based on the following three factors.

• Kasserine is the least wealthy region, in terms of average income per capita with the highest unemployment rate.
• Regional health staff and managers are highly motivated and supportive of the project objectives and to implement the project.
• Kasserine is in central Tunisia with extreme climatic conditions. Flat open plains to the south offered an ideal opportunity to demonstrate the project in difficult climatic conditions.

In addition to the municipality of Kasserine, three rural districts in the south of the Kasserine region were selected: Hassi Elfrid, Foussana and Feriana. The choice of districts was based on favourable solar irradiance and road infrastructure to implement the pilot project successfully. The three districts were finally filtered for data quality and a single district, Foussana, was selected to represent all three. Foussana had the most complete and correct data set of the districts and corresponded well with mean values for energy consumption in the three district data sets.

### 2.2 Baseline assessments

In order to compare results before and after the demonstration, baseline energy assessments were conducted [5]. In January 2010, the project team audited energy consumption at each participating regional and district facility, reporting on six components that use energy in the vaccine supply chain: refrigerators used to store vaccines; the vehicles for transporting vaccines; and the key components used at the storage facility (lighting, heating, air-conditioning and computer equipment including printers). The energy audits at each of the four demonstration sites provided a baseline consumption measure for each of the six elements. These energy estimates were converted [6] to their carbon emission equivalents at the rate of 2.7 kg CO_2_ per litre of fuel, 1.64 kg CO_2_ per kWh of grid (STEG) electricity and 0.43 kg CO_2_ per kWh of solar generated electricity. To standardize the metrics for consumption of electricity (kWh) and consumption of fuel (l), they were each converted at current energy prices and currency exchange for Tunisia and presented as a single, annual cost in US$.

### 2.3 Adaptations to the demonstration sites

Three modifications were made to the system to distribute vaccines and public health medicines in the project areas.

#### 2.3.1 Solar photovoltaic modules installed

Grid tied solar rooftop systems were installed on the roofs of the medical store at the regional directorate at Kasserine and in the three districts to meet the energy demand of vaccine storage facilities and transport (including electric vehicle charging, space cooling, refrigeration, lighting and computing). Excess energy generated was exported to the national grid but not reimbursed to the Ministry of Health^2^ according to thresholds set by the National Agency for Energy (ANME). Shortfalls of solar energy were automatically compensated by drawing energy from the grid. The maximum energy generated by these photovoltaic arrays was 15.84 kWp at Kasserine and 7.26 kWp at each district [7], designed to reflect the different levels of energy consumption at regional and district levels. The installations at all sites were inspected and certified by the ANME. The recorded production of energy of the photovoltaic arrays on each site is presented in the results.

#### 2.3.2 Reducing energy consumption

Energy consumption was minimized by changing the choice of equipment and transport (see Table 1). Transport consumed the greatest amount of energy of all categories within the distribution system at both the regional and district levels conventional diesel and petrol-powered vehicles have a much lower efficiency than all-electric vehicles. The fuel for each 4WD diesel-powered vehicle cost 5.83 US$/100 km more than the energy cost of the project electric vehicles. To minimize consumption of energy and convert to a renewable energy source, the project chose to purchase and use electric vehicles^3^ for all deliveries of vaccine and medicines and for supervision. The rationale for this choice was that by powering the vehicles from the same grid-linked solar electricity source as the stores, energy costs relative to diesel vehicles would be substantially reduced. The vehicles selected have a daily autonomy (kilometers available from one over-night charge) that is limited to the capacity of their battery bank. The region used 32% of the electric vehicles’ maximum autonomy of 145 km for trips of all purposes. The district vehicles were utilized within a range of 29–57% of their autonomy of 100 km. The range limitations of the electric vehicles were respected when planning supply delivery circuits and they were rarely a constraint. When a destination was too far or roads were too difficult, four-wheel drive diesel vehicles remained in use.

#### 2.3.3 Design of the distribution system

The demonstration in Kasserine modified the distribution of vaccines and certain medicines that were managed by the Public Health Regional Directorate in two ways. First, vaccines and medicines that were stored separately before the project were relocated under one roof and were delivered at the same time, using in the same vehicle. Second, the previous practice of mixed collection and delivery trips was changed to ‘deliveries-only’ using optimized delivery circuits and combining supply with supervision. This system is based on similar trials in Africa [8] and South-East Asia [9] that show the system to be efficient and enhanced both supply and the quality of supervision. Standard operating procedures were established to implement the system using electric utility vehicles to carry supplies and health supervisory staff. The vehicle returned each evening for an overnight charge from the grid at the regional or district storage facility.

### 2.4 Monitoring and evaluation

**Fig. 1:**
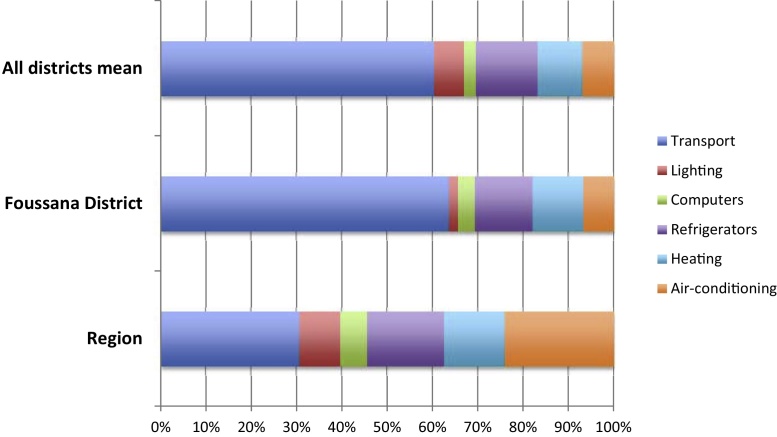
Percent distribution of estimated baseline annual energy costs (2010).

*Monitoring*: During the implementation of the project, certain monitoring systems were put in place to collect quantitative information needed to assess the NZE demonstration. Eight sensors were used to measure solar irradiance, ambient air temperature, power produced, electricity consumed in up to five electrical circuits including electric vehicle charging, space cooling, refrigeration, lighting and computing. The data was transmitted via Internet in real-time to a central server that presented data and reports on a project website. The data on energy consumption and production for the 12-month period in 2012 was analyzed and compared against the baseline energy audit assessments. The monitoring system suffered from several failures that resulted in missing data in two of the three districts monitored. These included the omission of an output from an array of solar modules in the energy recording system in one district; many Internet failures due to storms and line failures at two districts. As a consequence of the loss of data due to these causes the district of Foussana was chosen to represent the results of the four districts. Baseline data on consumption in all districts (see Fig. 1) show that data for Foussana match well with the mean for all project districts.

*Evaluation*: Quantitative evaluation of vaccine handling and supply chain performance indicators was made before (2010) and at the end of the Optimize project to assess the supply chain system and the impact of the interventions. This evaluation followed the method of the effective vaccine management (EVM) assessment tool of WHO [10]. The assessment measures supply chain performance in nine categories, with a target score of 80% for each. The 2010 assessment was conducted as a baseline at a sample of sites, and included the national vaccine stores (Central Pharmacy of Tunisia and the Directorate of Basic Health Care; Tunisia), 9 regional stores, 17 district stores, and 14 health centres. According to the critical indicators for each category, the criteria that scored the lowest were temperature control (E2), vaccine management (E8), stock control (E5), and transport (E6). The evaluation tool was used in 2010 [11] to create a baseline performance and again in 2012 [12] to measure impact in project zones.

In addition to the quantitative analysis, qualitative interviews were conducted [13] to gauge perceptions and appreciation of the NZE demonstration. One manager and eight implementers were interviewed [14] regarding the acceptability and feasibility of the net-zero energy supply chain demonstration.

## 3 Results

### 3.1 Baseline energy consumption assessment

The energy consumption audits (electricity and fuel) for the Kasserine regional store and the Foussana district store in 2010 were converted, as described in the previous section, into annual energy costs for storage and transport of vaccines and medicines at 2012 rates (see Fig. 1).

Fig. 1 shows the percent distribution of the total annual energy cost in 2010 for storage and transport in the district store at Foussana (1074 US$). The mean for all districts was 874 US$ and the regional store at Kasserine had the highest energy cost at 1679 US$. The costs of transporting vaccines and medicines were the greatest proportion of the total, followed by the combined cost of heating, air-conditioning and refrigeration. However, the consumption of air conditioning in all districts, including Foussana, was low at the baseline reflecting little use of this equipment. Some were clearly out of order and no longer in use but for others, the reasons were not clear.

### 3.2 Impact of NZE system on energy costs

**Fig. 2:**
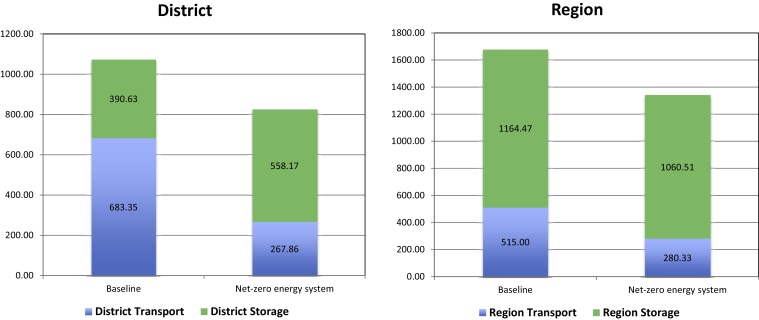
Energy consumption (supply chain energy costs only) at region and district (Foussana) levels expressed as cost at baseline and during net-zero energy intervention, 2012.

Compared to the baseline, the combined annual energy consumption for storage and transport using the net-zero energy system was reduced by 20.16% at the regional level and by 20.50% at the district level (see Fig. 2). Only storage consumption appear to increase (50%) at district level with the NZE modifications. This is due to increased use of heating, air conditioning, lighting and refrigeration when vaccines and medicines were relocated to an improved store under the same roof. Prior to NZE, transport accounted for 60% (district average) of total energy costs. Therefore the majority of savings came from switching four-wheel-drive diesel and petrol vehicles to electric vehicles on most delivery circuits for medicine and vaccine distribution.^4^

The electrification of transport brought two significant efficiency benefits; the first was due to an attribute of the technology and the second is due to changes in the management of distribution.

**Fig. 3:**
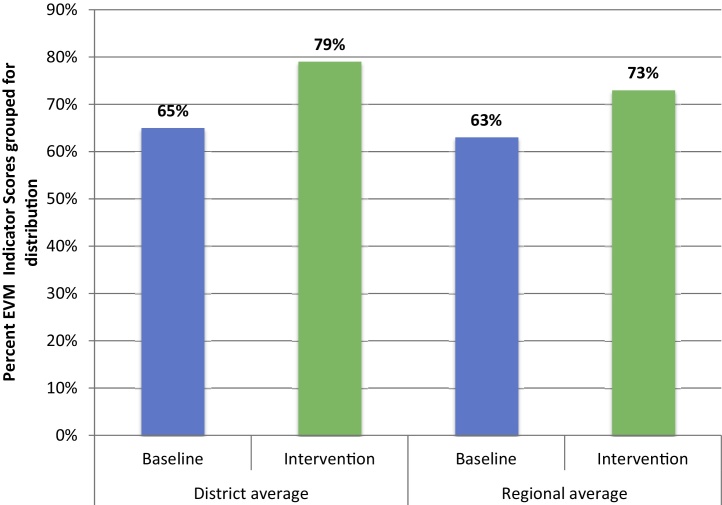
EVM distribution indicator scores – baseline (2010) versus intervention (2012). (Indicators combined for distribution performance. District mean values obtained from Foussana, Feriana and Hassi Elfrid districts.)

• *Technological*: The energy costs per 100 km for the project electric vehicles was 2.71 US$ based on the dollar value of the electricity consumed by nightly charging of the vehicles. This cost is already 69% less than the fuel cost of the 4WD vehicles used at regional an district levels in Kasserine. Even when compared to the same size of 2WD diesel powered vehicle, the saving would be 54%. But because in the NZE project the energy used to charge the vehicles is supplied by the grid-linked solar powered system, there are no recurrent costs of energy for transport of vaccines, medicines and for supervision.
• *Managerial*: The scheduling of fixed delivery circuits for vaccines and medicines minimized the distances travelled by the electric vehicles and avoided costly ‘emergency’ journeys to collect vaccine. There were also benefits from integrating storage and distribution of vaccines and medicines [15]. The best general indicator of the improved efficiency of the distribution system achieved during the project interventions including the use of dedicated electric vehicles was the improvement of performance indicator scores from the ‘effective vaccine management’ (EVM) assessments conducted as a baseline in 2010 and the subsequent assessment in 2012. Fig. 3 combines a selection of EVM indicators that measure the performance of distribution at district level to health centres and at regional level to district stores. The figure shows significant improvement at both levels.

### 3.3 NZE consumption vs. production energy balance

**Fig. 4:**
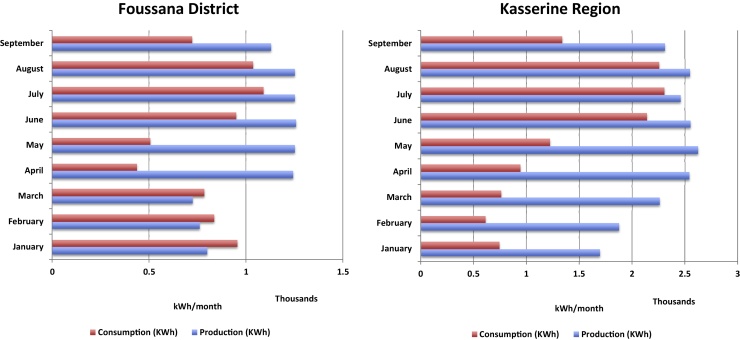
Monthly energy production compared to energy consumption.

Fig. 4 compares the monthly energy production to the monthly consumption at the Kasserine regional store and the Foussana district store. The results show that at the regional level, production exceeded consumption in all months. At the district store of Foussana production exceeded consumption overall except in January to March when consumption exceeded production. In months of energy surplus the system automatically offset consumption in the main Ministry of Health buildings on the site of the store.

Together, Kasserine regional store and Foussana district store showed a positive energy balance during 2012 (15,537 kWh), meeting the project goal of net-zero energy by producing the same or more renewable energy than was consumed for vaccine storage and transport. Overall, including storage and transport, NZE achieved a 67% drop in the release of carbon to the atmosphere.

### 3.4 Qualitative assessment of health staff opinions

All health staff participating in the assessment (9/9) reported that the NZE intervention was appropriate, and eight participants (8/9) declared that the intervention was acceptable or very acceptable. Positive feedback was provided on the implementation of electric vehicles, the installation of solar panels and the offset of energy consumption. Respondents reported that the accessibility of the electric vehicles dedicated to delivery of vaccines and medicines increased the reliability and efficiency of distribution and improved vaccine handling.

## 4 Discussion

This project set out to show the impact of applying the net zero energy principles to the storage and transport of vaccines and medicines in the context of other selected managerial interventions to streamline the current system. The project has demonstrated the impact of net zero energy on energy consumption and energy cost. It has also demonstrated the combined impact of several interventions, including net zero energy on supply chain performance. Without attributing this impact directly to net zero energy, the combined results show an improvement measured against the baseline (EVM assessment indeces).

Application of the net zero energy concept to the energy needs of storage and transport of vaccines and medicines offers several benefits even in the absence of other interventions:

• Using energy audits to detect and eliminate energy waste.
• Using grid-linked solar energy generation to offset non-renewable energy requirements including both transport and storage.
• Reducing recurrent energy costs of distribution thus freeing health budgets to expand service provision.
• Achieving significant reduction in carbon ‘footprint’.

In addition, the net zero energy intervention is highly compatible with managerial interventions to streamline the supply chain for vaccines and medicines by:

• Establishing a delivery-based distribution, uniting supply and supervision on reliable schedule at minimal energy cost.
• Raising the standard of sustainability in the system by integrating the supply chain for vaccines and medicines.

The missing element in the rationale for scale-up of net zero energy and a limitation of this study is a credible economic argument setting the necessary capital investment against whole-life gains in recurrent expenditure on energy. Such an analysis would take account of the decline in the cost of solar energy and the prospects for electric vehicle service infrastructure. Nevertheless, the project recommended that the first steps, of universal benefit, be taken towards net zero energy in parallel to further rationalization, including:

• Accelerate existing programme of energy auditing of vaccine stores and making improvements to building insulation, lighting, refrigeration, and informatics equipment to minimize energy consumption.
• Implement grid linked solar photovoltaic energy for Ministry of Health warehousing of drugs and vaccines, extending the current national policy in support of domestic grid-linked projects.
• Conduct study of the electric vehicle market (including hybrids), facilities for maintenance in country, and prioritization of requirements.

## Figures and Tables

**Table 1:** Optimize project Interventions to reduce energy consumption.

Category	Baseline (2010)	Optimize intervention (2012)
Transport	Petrol/diesel four-wheel-drive vehicles used for all purposes	All-electric vehicles used for vaccine and medicines distribution
	Freeze water-icepacks to −20 °C to cool vaccines in transport using picnic coolers	Refrigerate PCM-packs to +2 °C to cool vaccines in transport using high performance cold-boxes

Lighting	Fluorescent tubes and incandescent lamps	LED-based tubes and lamps
Computer equipment	Desktop computers	Laptop computers
Refrigerators	Domestic refrigerators (energy class 4 and 5)	Domestic refrigerators (energy class 2 and 3)

## Footnotes

1 Region of Kasserine, the Municipality and the Districts of Foussana, Feriana and Hassi Elfrid.

2 The national grid authority ‘STEG’ (Société Tunisienne de l’Electricité et du Gaz) did not, at the time of this project and at this level of production, credit surplus energy to the electricity account of the Ministry of Health.

3 Fiat Fiorino/Microvet, Monaco.

4 Some health centres were on roads unsuitable for electric vehicles; so four-wheel-drive vehicles were used in those circumstances.
